# Content accuracy and reliability of pulmonary nodule information on social media platforms: a cross-platform study of YouTube, Bilibili, and TikTok

**DOI:** 10.3389/fmed.2025.1613526

**Published:** 2025-09-22

**Authors:** Yunanji Zhou, Xiang Zeng, Ting Yuan, Qian Wang, Siqi Wu, Lei Du, Lihua Wang, Jun He

**Affiliations:** ^1^Qi Huang Chinese Medicine Academy, Jiangxi University of Chinese Medicine, Nanchang, China; ^2^School of Clinical Medicine, Jiangxi University of Chinese Medicine, Nanchang, China; ^3^Pulmonary Disease Department, Affiliated Hospital of Jiangxi University of Traditional Chinese Medicine, Nanchang, China

**Keywords:** pulmonary nodule, public health, social media, online video, information quality

## Abstract

**Background:**

Pulmonary nodules (PNs) are often overlooked, potentially leading to health risks. Social media platforms are increasingly used for health information dissemination. This study evaluates the quality and engagement of PN-related videos on YouTube, Bilibili, and TikTok.

**Methods:**

On March 1, 2025, we searched each platform using “pulmonary nodule” or its Chinese equivalent. After screening, 271 videos were analyzed. Video characteristics were documented, and quality was assessed using PEMAT, VIQI, GQS, and mDISCERN tools. Inter-rater reliability was high (*κ* = 0.81).

**Results:**

The final sample included 98 (YouTube), 74 (Bilibili), and 99 (TikTok) videos. TikTok videos were the shortest (median 114 s) yet had the highest engagement. Nonprofit organizations dominated YouTube uploads; physicians were most common on Bilibili and TikTok. Treatment was the most covered topic. YouTube scored highest in comprehensibility and actionability (PEMAT-T/A), while Bilibili and TikTok scored higher in production quality (VIQI, GQS). Video quality did not differ significantly between professional and non-professional uploaders. Most quality metrics showed weak correlation with audience engagement.

**Conclusion:**

Long-form platforms (YouTube and Bilibili) offer higher-quality PN information but lower engagement, whereas short-form platforms (TikTok) show high interaction but lower informational depth. Social media can play a supportive role in public PN education. We provide recommendations for creators, platforms, and viewers to improve the quality and reliability of medical content.

## 1 Introduction

A pulmonary nodule (PN) is a localized, rounded, high-density shadow observed on chest imaging (e.g., computed tomography [CT] or X-ray), typically measuring less than 3 cm in diameter with well-defined or hazy borders (1). Based on density, PNs are classified into three categories: solid nodules, part-solid nodules, and pure ground-glass nodules (2). The majority of benign solid nodules are associated with tuberculosis (TB) or inflammatory conditions (3). Malignant solid nodules can be precursors to lung cancer. Malignancy should be suspected in solid nodules exhibiting features such as spiculation, lobulation, or irregular margins (4). Part-solid nodules, also termed mixed ground-glass nodules, contain both solid and ground-glass components and exhibit heterogeneous density on CT (2). These nodules have a relatively high malignancy probability, particularly when the solid component increases, which often indicates progression (1). This often signifies early-stage lung cancer, necessitating prompt management and close monitoring (5). After follow-up, most part-solid nodules may resolve spontaneously or remain stable. However, persistent or growing nodules with increasing density carry a high malignancy risk (6).

Clinically, identifying individuals with lung nodules is challenging due to the frequent absence of overt symptoms. The detection rate of asymptomatic lung nodules has increased dramatically in China, largely due to the widespread adoption of low-dose CT screening, which offers substantial potential for the early diagnosis of lung cancer (7). Early and accurate diagnosis of lung nodule type is crucial for improving patient prognosis and reducing lung cancer mortality, as lung cancer remains a leading cause of cancer-related death worldwide (8). Consequently, improving public understanding of the various types of lung nodules and the distinctions between benign and malignant forms is critical.

Social media platforms have become increasingly popular for sharing and exchanging knowledge. YouTube is the dominant video platform in international markets (9), whereas TikTok and Bilibili are the most popular in China (10, 11). Video-based learning about current events and knowledge has gained popularity, and videos play a significant role in health communication and intervention. However, video quality varies considerably due to diverse content sources and differing levels of platform content regulation. A recent report indicated that the top 100 COVID-19 videos on TikTok garnered over 1.19 billion views, yet the most frequently discussed topics were not the most relevant to public health information (12). This suggests that videos under pertinent keywords may disseminate undesirable emotions and often lack scientific or expert medical knowledge. Moreover, the motives behind physicians’ sharing of health information, which can range from altruism to financial incentives, significantly impact the quality and reliability of content (13). Understanding these dynamics is crucial for framing effective platform policies and incentive structures. This study aims to assess and analyze PN-related videos on YouTube, Bilibili, and TikTok with the goals of raising public awareness and providing actionable recommendations to platforms for enhancing the reliability of medical content.

Clinically, it is challenging to identify people with lung nodules because they typically do not exhibit any overt symptoms. The detection rate of asymptomatic lung nodules has dramatically grown in recent years due to China’s extensive use of low-dose CT screening, which offers a substantial potential for the early diagnosis of lung cancer (7). Early and accurate diagnosis of the kind of lung nodules is crucial for improving patient prognosis and lowering lung cancer mortality, as lung cancer is one of the world’s leading causes of cancer-related fatalities (8). As a result, it is critical that the general public understands the many kinds of lung nodules and the differences between benign and malignant ones.

## 2 Method

### 2.1 Search strategy

Before collecting video feature information, we searched for “pulmonary nodule,” “lung nodule,” “solitary pulmonary nodule,” “ground glass nodule,” and “coin lesion” on three platforms and found that all search results were similar to those obtained using only “pulmonary nodule.” Furthermore, using “pulmonary nodule” yielded the highest number of search results. On March 1, 2025, we searched on YouTube using the English keyword “Pulmonary Nodule,” and on Bilibili and TikTok using the term “肺结节” (“Pulmonary Nodule” in Chinese).

To minimize the bias of the platforms’ algorithmic recommendations, we created new accounts and deleted browser caches and search histories. The included videos were required to be posted by February 15, 2025. The top 100 videos for each platform were retrieved without the use of filtering criteria, and the results are shown in “default order” across all platforms. Due to the regional applicability of the three video platforms, we found that almost all non-English/Chinese videos were reposted (not original) from existing publishers. Videos featuring commercials, videos in languages other than English and Chinese, videos regarding infants and young children, duplicates, and unnecessary videos were all disqualified (Supplementary file 1). One individual gathered and downloaded all of the videos; two investigators classified the categories of videos and the uploaders. If discrepancies arose, the authors collectively reviewed the different videos to determine whether to retain them or not.

### 2.2 Video content

A number of video attributes, including length, duration, views, thumbs up, comments, collections, shares, coin-operated, upload time, upload source, author, and video attributes, were methodically recorded by two investigators on the same day (Supplementary file 1). However, for (1) TikTok views and (2) YouTube collections and shares, the following data were not accessible. The uploader’s ID, number of followers, authentication status, and kind are among the many details gathered. Using preset criteria, professionals and credentials were located (Supplementary file 1). Videos in rough form, translations, or direct copies are not considered original. The many forms of video filming include solo narration, Q&A, PPT or class, animation/action, and medical scenarios.

### 2.3 Video review and classification

Two researchers looked over the listed videos independently between March 3 and 5, 2025, removing a large number of videos that were identical or unrelated (Supplementary file 1). The topics covered in the videos were etiology/prevention, anatomy, pathology, epidemiology, symptoms, exams, diagnosis, and prognosis. The number of subjects each video addressed was counted since several movies were relevant to the broad spectrum of issues. Videos that did not cover these topics were judged superfluous and were taken off.

### 2.4 Video quality assessment

Video characteristics were systematically recorded. Two independent reviewers classified uploader type and video content. Between March 6 and 9, 2025, two respiratory disease specialists performed a double-blind evaluation of video quality using four validated tools: PEMAT, VIQI, GQS, and mDISCERN. The inter-rater reliability was excellent (Cohen’s *κ* = 0.81). Disagreements were resolved through discussion or by a third senior expert. All three respiratory disease specialists have over 10 years of clinical experience and extensive involvement in health education (e.g., publishing articles, recording educational videos, and providing educational services in schools and communities).

The research team used Cohen’s kappa coefficient (*κ*) for reliability analysis to measure the degree of inter-assessor agreement. Its determination criterion was based on international norms: κ > 0.8 indicates excellent agreement, 0.6–0.8 indicates good agreement, 0.4–0.6 indicates moderate agreement, and ≤0.4 indicates insufficient agreement (14).

The Modified DISCERN Scale (mDISCERN) was used to evaluate the information’s credibility (15), the Global Quality Scoring System (GQS) concentrates on the overall production standards (16), the Video Information Quality Index (VIQI) examines the content’s quality from a multidimensional perspective (16), and the Patient Education Materials Assessment Tool (PEMAT) focuses on the comprehensibility and actionability of health education materials (17) (Supplementary file 2). It is important to note that these evaluation instruments have demonstrated their reliability in a number of worldwide studies and have a wide range of uses, particularly in the area of social media health information assessment (15–18).

### 2.5 Statistical analysis

The normality of continuous variables was assessed using the Shapiro–Wilk test. Due to the non-normal distribution of the data, descriptive statistics are presented as median (min-max) and interquartile range [IQR] (P25, P75). Group comparisons for non-normally distributed continuous variables were performed using the Mann–Whitney U test. Categorical variables were compared using Chi-square tests, with continuity correction or Fisher’s exact test applied as appropriate. Correlations between quantitative variables were assessed using Spearman’s rank correlation coefficient. A two-sided *p*-value of < 0.05 was considered statistically significant. For Spearman’s correlation, a positive association was indicated by *r* > 0, and a negative association by *r* < 0. The strength of the link was classified. All statistical analyses were performed using R software (version 4.4.2; R Foundation for Statistical Computing).

## 3 Results

### 3.1 Video characteristics

**Figure 1:**
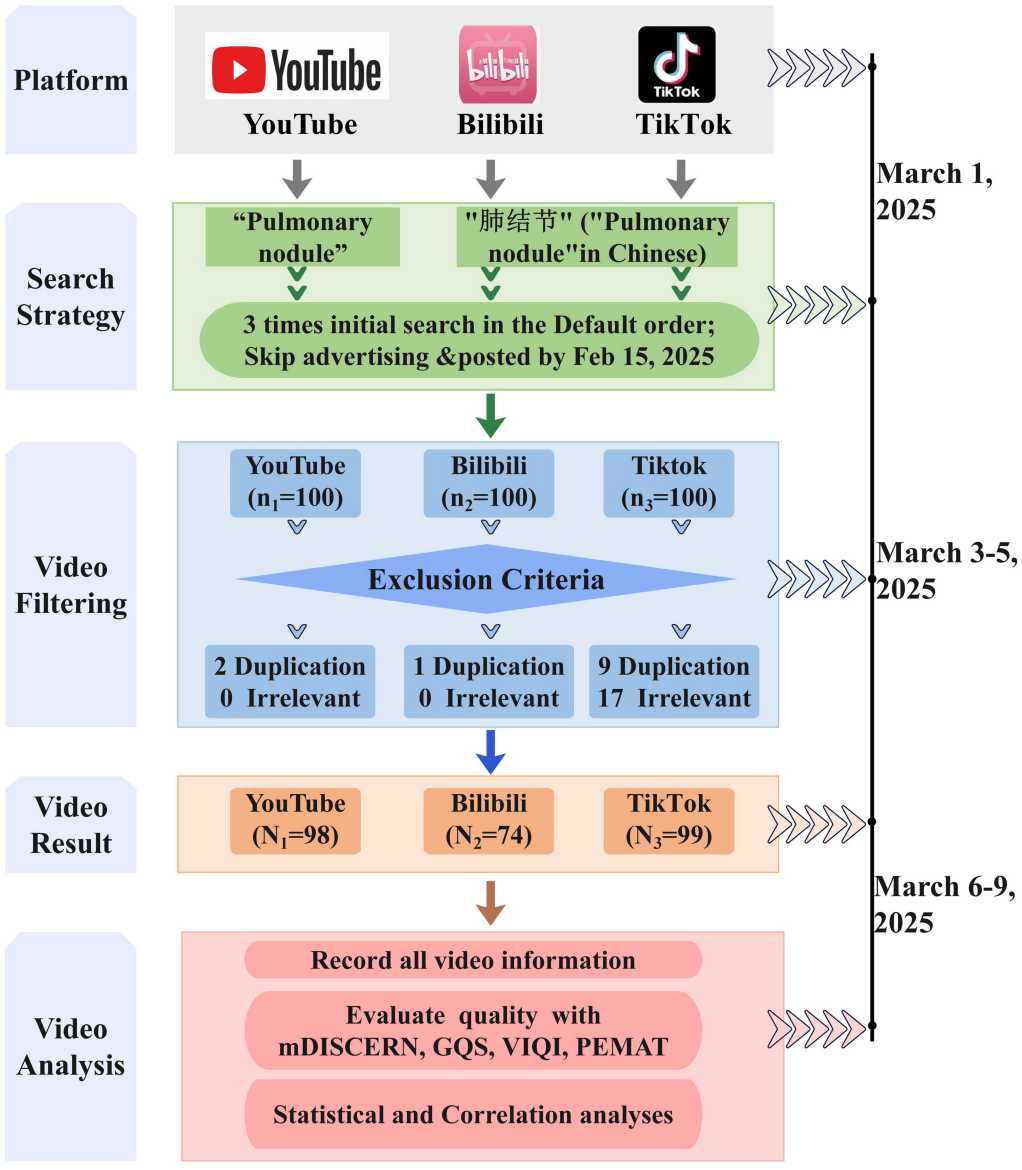
Search strategy for videos on pulmonary nodule.

**Figure 2:**
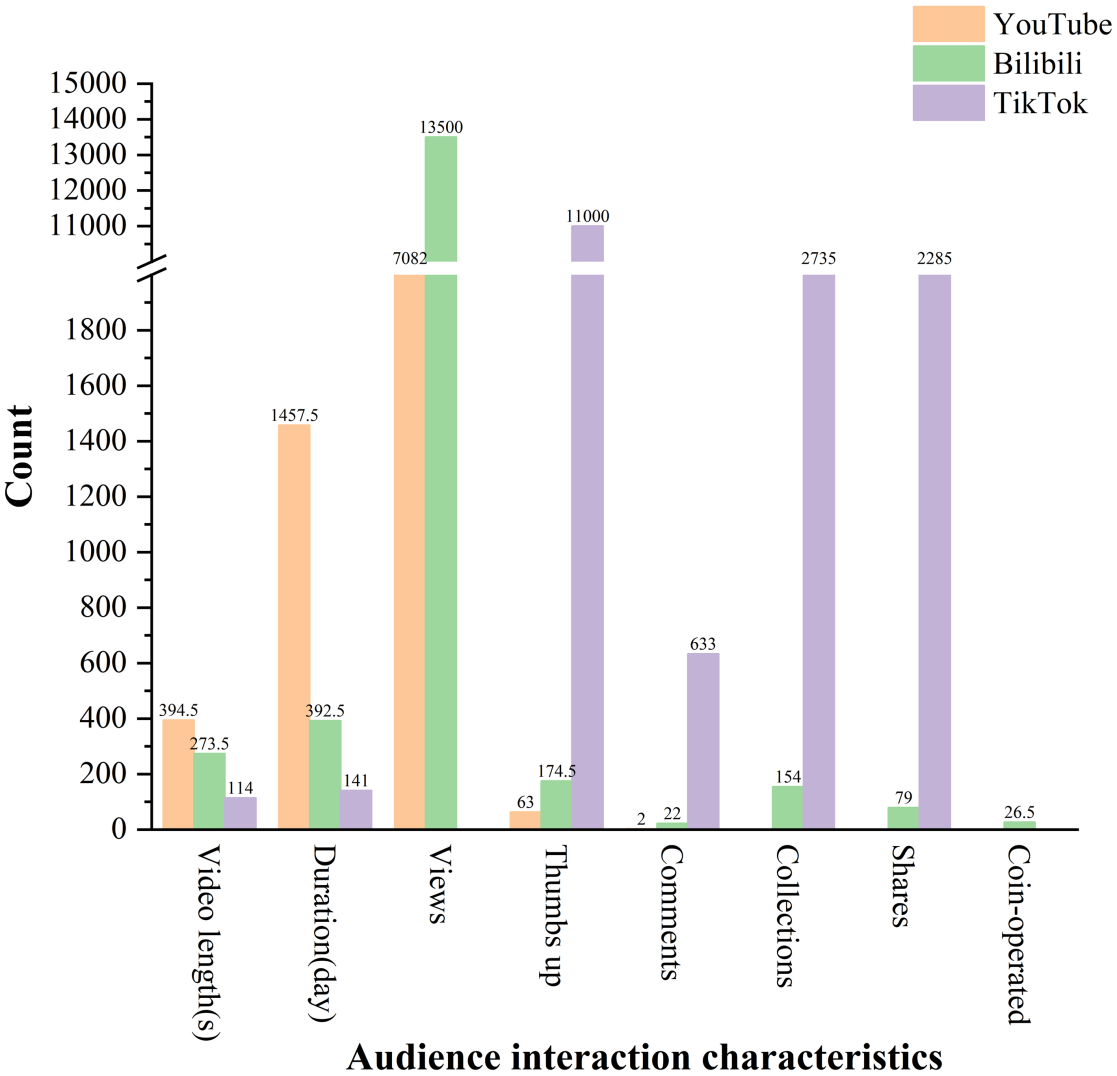
Characteristics of video viewer interaction on different platforms (mean).

After removing duplicates and irrelevant content, the final dataset comprised 98 YouTube videos, 74 Bilibili videos, and 99 TikTok videos (Figure 1). All videos across these platforms were either in Chinese or English or provided bilingual subtitles. The Shapiro–Wilk test revealed that all continuous variables exhibited non-normal distributions. Table 1 and Figure 2 provide specific details about the videos from each platform. Notably, TikTok videos (114 [19–1,321] seconds) were significantly shorter than those on YouTube (394.5 [26–3,743] seconds) and Bilibili (273.5 [54–3,983] seconds). TikTok also demonstrated more frequent updates based on release dates. TikTok had the most likes and comments out of the three sites. YouTube displayed the least amount of interaction from users, whereas Bilibili had the most extensive interactive components.

**Table 1:** Characteristics of video about laryngeal carcinoma on YouTube/ Bilibili/ TikTokk.

Characteristic	YouTube (N_1_ = 98)			Bilibili (N_2_ = 74)			TikTok (N_3_ = 99)			*p*-value		
	*M*	Min-Max	P25-P75	*M*	Min-Max	P25-P75	*M*	Min-Max	P25-P75	P_(Y-B)_	P_(B-T)_	P_(Y-T)_
Video length(s)	394.5	26–3,743	117.75–1,177	273.5	54–3,983	134.5–466.5	114	19–1,321	70–205.5	0.115	**<0.001**	**<0.001**
Duration(day)	1457.5	169–5,621	755–2608.25	392.5	1–1,573	288.25–771	141	1–1,058	51–315.5	**<0.001**	**<0.001**	**<0.001**
Views	7,082	46–695,780	1,631–25153.25	13,500	193–357,000	2452.25–40,500	-	-	-	0.122	-	-
Thumbs up	63	0–5,803	7–252.5	174.5	5–15,000	55–787.75	11,000	29–450,000	2254.5–28,000	**<0.001**	**<0.001**	**<0.001**
Comments^a^	2	0-674	0-25.5	22	0–1,073	5–104	633	1–17,000	144.5–1,642	**<0.001**	**<0.001**	**<0.001**
Collections	-	-	-	154	3–5,921	45–668.5	2,735	7–214,000	543.5–12,000	-	**<0.001**	-
Shares	-	-	-	79	3–3,361	18.5–456	2,285	2–188,000	396–7,037	-	**<0.001**	-
Coin-operated	-	-	-	26.5	0–1,319	7–125.75	-	-	-			

All the *p*-values were obtained from Mann–Whitney U test.

P_(Y-B)_: YouTube versus Bilibili; P_(B-T)_: Bilibili versus TikTok; P_(Y-T)_: YouTube versus TikTok. Bold text means the *p*-value < 0.05.

a Excluded: 16 videos on YouTube turned off the function of comments.

### 3.2 Uploader profiles and certification status

**Figure 3:**
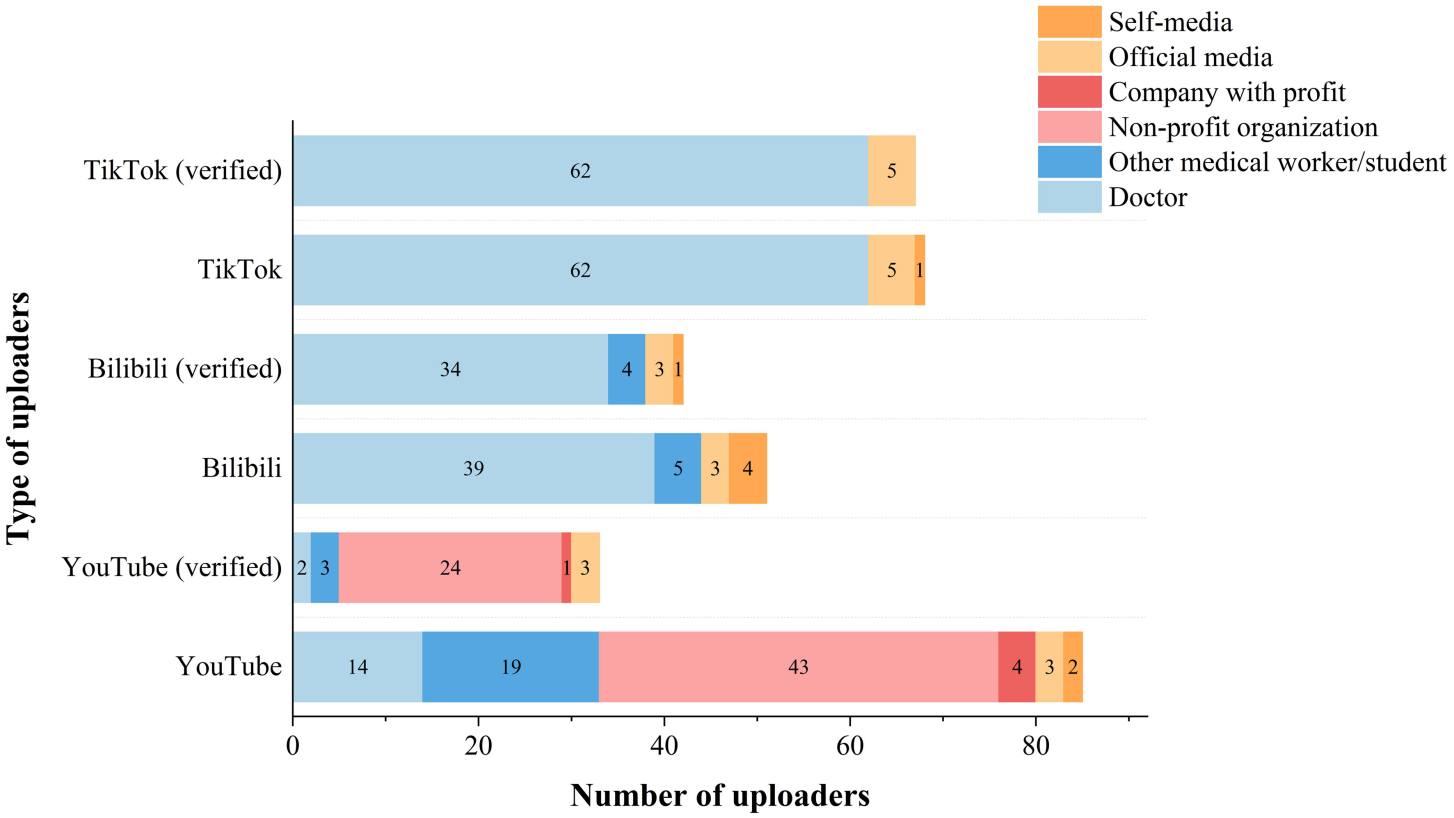
Number of video uploaders by different platforms.

This research video included 85 YouTube uploaders, 51 Bilibili uploaders, and 68 TikTok uploaders. The categories of uploaders on different platforms vary greatly (Table 2; Figure 3). Doctors account for 91.2% on TikTok, 76.5% on Bilibili, and only 16.5% on YouTube. Non-profit organizations accounted for 50.6% of uploaders on YouTube, but were absent on Bilibili and TikTok. Bilibili uploaders posted videos more regularly than the less active YouTubers. The certification rates were 38.8% on YouTube, 82.4% on Bilibili, and 98.5% on TikTok. We examined certified uploaders to determine the video’s authority. Only a small percentage of YouTube-certified doctors were included in this study, despite having the highest subscribers. Furthermore, 15 acknowledged Traditional Chinese Medicine (TCM) physicians posted to TikTok, and 21 recognized TCM physicians uploaded to Bilibili. Among the videos, one titled ‘Heart Art’ received a high number of likes.

**Table 2:** Characteristics of video uploaders about laryngeal carcinoma on YouTube/ Bilibili/ TikTok.

Platform	YouTube	Bilibili	TikTok	P_(Y-B)_	P_(B-T)_	P_(Y-T)_
Number of uploaders	85	51	68	-	-	-
Followers, median [P25, P75]	10,000 [1880, 52,600]	29,000 [3,605, 75,750]	269,000 [68,750, 1,048,750]	0.079^a^	<**0.001**^a^	<**0.001**^a^
Number of videos per person, Mean±SD, Median[P25, P75]	1.15 ± 0.42, 1 [1, 1]	1.48 ± 0.93, 1 [1, 2]	1.46 ± 1.20, 1 [1, 1]	0.021^a^	0.328^a^	0.215^a^
Type of uploaders, *n* (%)						
Doctor	14 (16.5%)	39 (76.5%)	62 (91.2%)	<**0.001**^b^	**0.027** ^**b**^	<**0.001**^b^
Other medical worker/student	19 (22.4%)	5 (9.8%)	0	0.063^b^	**0.030** ^**c**^	<**0.001**^b^
Non-profit organization	43 (50.6%)	0	0	<**0.001**^b^	-	<**0.001**^b^
Company with profit	4 (4.7%)	0	0	0.294^c^	-	0.193^c^
Official media	3 (3.5%)	3 (5.9%)	5 (7.4%)	0.829^c^	1.000^c^	0.490^c^
Self-media	2 (2.3%)	4 (7.8%)	1 (1.4%)	0.281^c^	0.210^c^	1.000^c^
Doctor of TCM, *n* (%)	0	21 (41.2%)	15 (22.1%)	<**0.001**^b^	**0.025** ^**b**^	<**0.001**^b^
Authentication, *n* (%)	33 (38.8%)	42 (82.4%)	67 (98.5%)	<**0.001**^b^	**0.005** ^**c**^	<**0.001**^b^
Verify the type of uploader, *n* (%)						
Doctor	2 (6.1%)	34 (81.0%)	62 (92.5%)	<**0.001**^b^	0.069^b^	<**0.001**^b^
Other medical worker/student	3 (9.1%)	4 (9.5%)	0	1.000^c^	**0.040** ^**c**^	0.034^d^
Non-profit organization	24 (72.7%)	0	0	<**0.001**^b^	-	<**0.001**^b^
Company with profit	1 (3.0%)	0	0	0.440^d^	-	0.330^d^
Official media	3 (9.1%)	3 (7.1%)	5 (7.5%)	1.000^c^	1.000^c^	1.000^c^
Self-media	0	1 (2.4%)	0	1.000^d^	0.385^d^	-

P_(Y-B)_: YouTube versus Bilibili; P_(B-T)_: Bilibili versus TikTok; P_(Y-T)_: YouTube versus TikTok. Bold text means the *p*-value < 0.05.

a Mann-Whitney U test.

b Chi-squared test.

c Continuity correction.

d Fisher’s exact test.

### 3.3 Content categories and presentation styles

**Figure 4:**
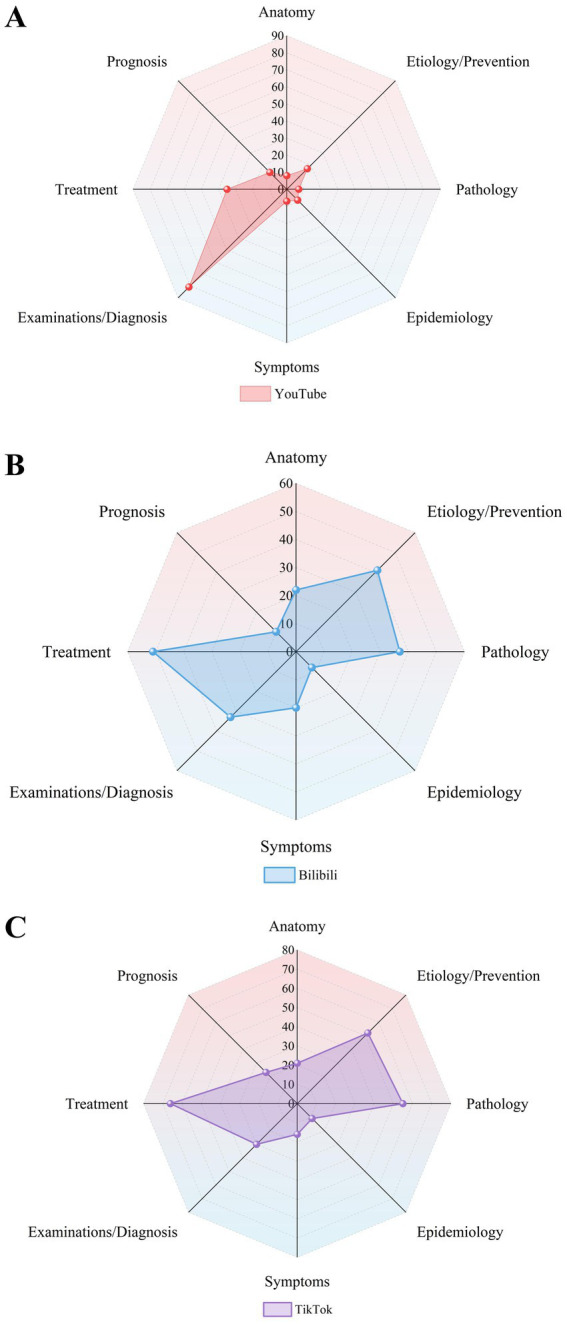
Types of topics for video content on different platforms. (**A** data source YouTube; **B** data source Bilibili; **C** data source TikTok).

**Figure 5:**
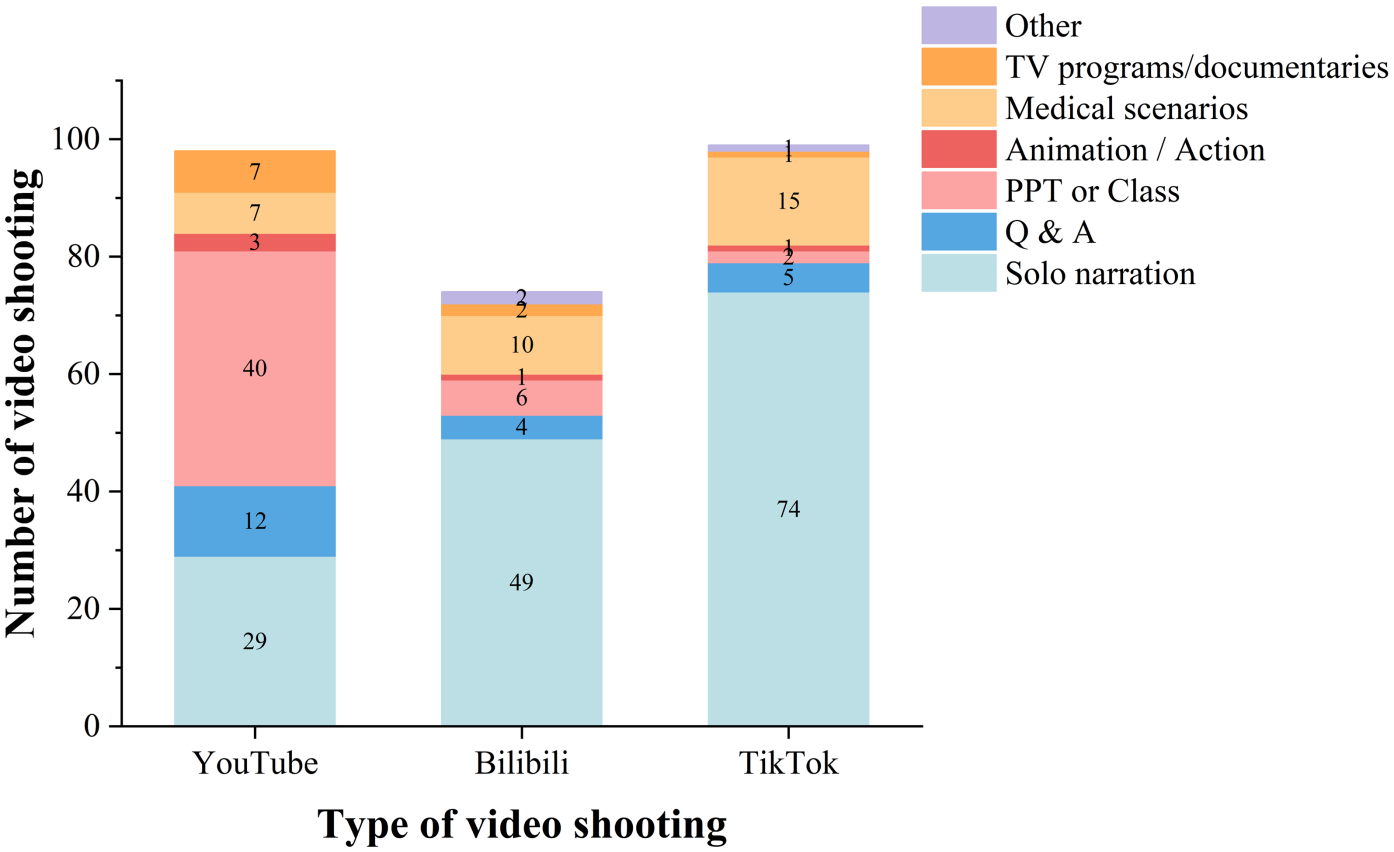
Various styles of platform videography.

Table 3 and Figure 4 describe the video categories. TikTok (100%) and YouTube (100%) contained more raw materials than Bilibili (97.3%). The videos on Bilibili cover a wider range of topics than those on YouTube and TikTok, indicating that there are differences in the diversity of video topics across different platforms. Interestingly, after a thorough review, we found that the number of topics is correlated with longer video lengths. The most viewed videos on all three platforms were those with therapeutic content. The difference is that TikTok and Bilibili were more interested in etiology/prevention and pathology, while the most popular topic on YouTube was examination/diagnosis. Treatment options for PNs were covered in a number of videos, all of which emphasized the significance of keeping a positive psychological attitude after being detected. According to several video presentations, the majority of PNs are benign, and the most commonly advised therapy strategy is routine follow-up exams. Certain medical experts added clinical case demonstrations using chest CT scans to their videos. Understanding anatomy and etiology has helped the public recognize the need for prevention. Platform-to-platform variations in content presentation patterns were substantial (Table 3; Figure 5). Solo narration predominated on TikTok and Bilibili, while PPT or class presentations were most prevalent on YouTube. In addition, TikTok is skilled at using medical scenarios.

**Table 3:** Categorization of videos about laryngeal carcinoma on YouTube/ Bilibili/ TikTok.

Platform	YouTube (N_1_ = 98)	Bilibili (N_2_ = 74)	TikTok (N_3_ = 99)	P_(Y-B)_	P_(B-T)_	P_(Y-T)_
Originality, *n* (%)	98 (100%)	72 (97.3%)	99 (100%)	0.184^d^	-	0.182^d^
Number of topics per video, Median [P25, P75]	1.5 (1–2)	3 (2–4)	2 (2–3)	**<0.001** ^**a**^	0.239^a^	**<0.001** ^**a**^
Type of topics, *n* (%)						
Anatomy	8 (8.4%)	22 (29.7%)	21 (21.1%)	**<0.001** ^**b**^	0.164^b^	**0.010** ^**b**^
Etiology/Prevention	17 (17.3%)	41 (55.4%)	52 (52.5%)	**<0.001** ^**b**^	0.567^b^	**<0.001** ^**b**^
Pathology	7 (7.1%)	37 (50%)	55 (55.6%)	**<0.001** ^**b**^	0.589^b^	**<0.001** ^**b**^
Epidemiology	9 (9.2%)	8 (10.8%)	11 (11.1%)	0.723^b^	1.000^b^	0.654^b^
Symptoms	7 (7.1%)	20 (27.0%)	16 (16.1%)	**<0.001** ^**b**^	0.066^b^	**0.049** ^**b**^
Examinations/Diagnosis	81 (82.7%)	33 (44.6%)	30 (30.3%)	**<0.001** ^**b**^	**0.038** ^**b**^	**<0.001** ^**b**^
Treatment	35 (35.7%)	51 (68.9%)	66 (66.7%)	**<0.001** ^**b**^	0.563^b^	**<0.001** ^**b**^
Prognosis	14 (14.3%)	10 (13.5%)	23 (23.2%)	0.885^b^	0.126^b^	0.108^b^
TCM, *n* (%)	0	30 (40.5%)	17 (17.1%)	**<0.001** ^**b**^	**<0.001** ^**b**^	**<0.001** ^**b**^
Style of video shooting, *n* (%)						
Solo narration	29 (29.6%)	49 (66.2%)	74 (74.7%)	**<0.001** ^**b**^	0.336^b^	**<0.001** ^**b**^
Q & A	12 (12.3%)	4 (5.4%)	5 (5.1%)	0.140^b^	1.000^c^	0.012^b^
PPT or Class	40 (40.8%)	6 (8.1%)	2 (2.0%)	**<0.001** ^**b**^	0.118^c^	**<0.001** ^**b**^
Animation / Action	3 (3.1%)	1 (1.4%)	1 (1.0%)	0.842^c^	1.000^d^	0.403^c^
Medical scenarios	7 (7.1%)	10 (13.5%)	15 (15.2%)	0.147^b^	0.818^b^	0.295^b^
TV programs/documentaries	7 (7.1%)	2 (2.7%)	1 (1.0%)	0.363^c^	0.780^c^	**0.022** ^**c**^
Other	0	2 (2.7%)	1 (1.0%)	0.178^d^	0.780^c^	1.000^d^

P_(Y-B)_: YouTube versus Bilibili; P_(B-T)_: Bilibili versus TikTok; P_(Y-T)_: YouTube versus TikTok. Bold text means the *p*-value < 0.05.

a Mann-Whitney U test.

b Chi-squared test.

c Continuity correction.

d Fisher’s exact test.

### 3.4 Quality assessment of videos

**Figure 6:**
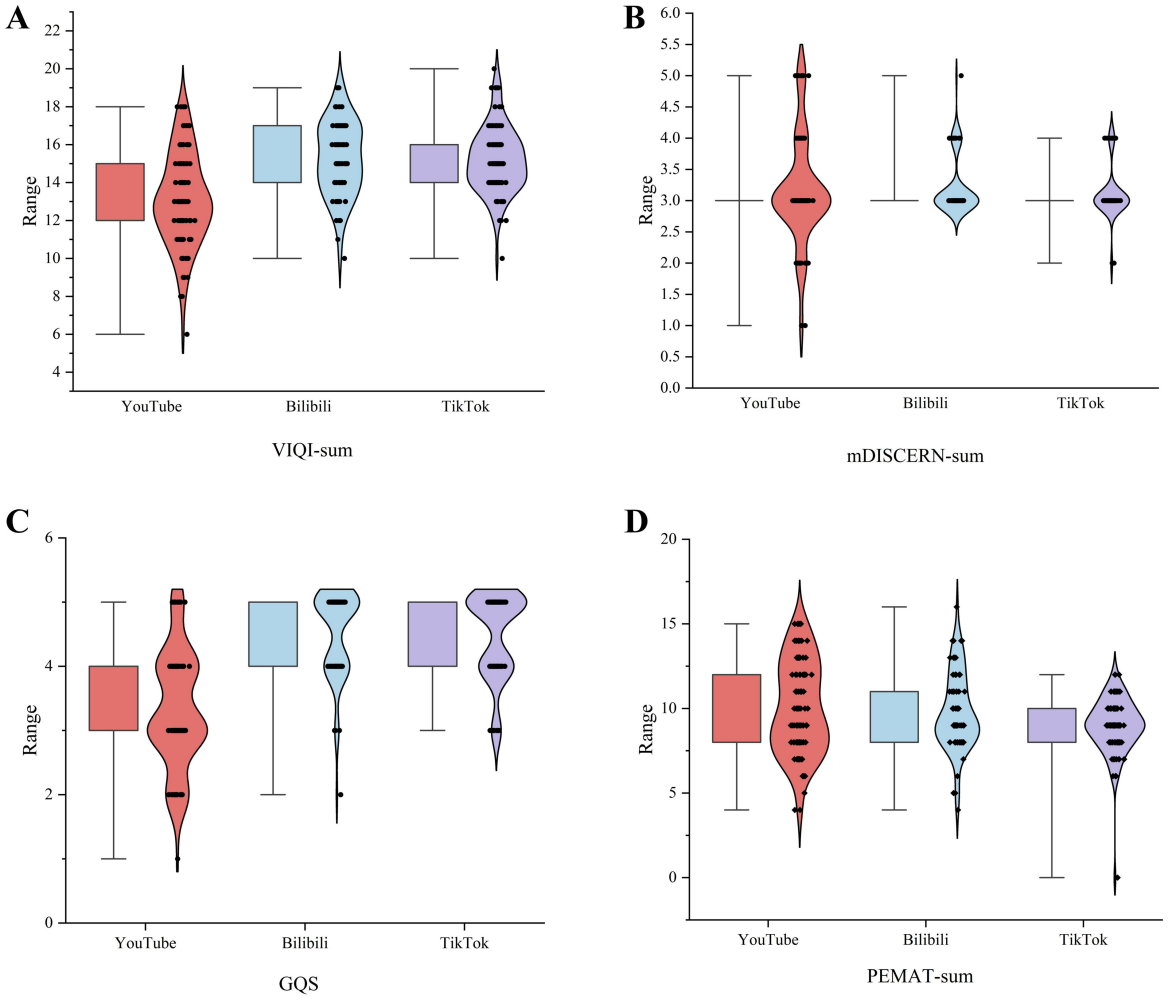
Quality assessment of videos about pulmonary nodule on YouTube/ Bilibili/ TikTok. (**A** for VIQI-sum score; **B** for mDISCERN-sum score; **C** for GQS score; **D** for PEMAT-sum score; red for YouTube; blue for Bilibili; purple for TikTok).

Inter-rater reliability was assessed using Cohen’s kappa, which indicated substantial agreement (*κ* = 0.81). The results of the video quality evaluation are presented in Table 4; Figure 6. YouTube demonstrated significantly higher PEMAT-T (comprehensibility) and PEMAT-A (operability) scores compared with Bilibili and TikTok (*p* < 0.001). In contrast, PEMAT-U (usability) ratings followed an inverse pattern. TikTok and Bilibili showed substantially higher VIQI and GQS ratings than YouTube (*p* < 0.001). No significant differences were observed in mDISCERN total scores (information reliability) across the three platforms. As shown in Table 5, no significant differences were found in PEMAT, VIQI, GQS, or mDISCERN scores between videos from professional and non-professional uploaders (*p* > 0.05).

**Table 4:** Quality assessment of videos about pulmonary nodule on YouTube/ Bilibili/ TikTok.

Platform characteristic	YouTube (N_1_ = 98)			Bilibili (N_2_ = 74)			TikTok (N_3_ = 99)			*p*-value		
	*M*	Min–Max	P25-P75	*M*	Min–Max	P25-P75	*M*	Min–Max	P25-P75	P_(Y-B)_	P_(B-T)_	P_(Y-T)_
PEMAT-T	9.5	4–15	8–12	9	4–16	8–11	9	6–12	8–10	0.787^a^	0.071^a^	**0.033** ^**a**^
PEMAT-U	3	2-4	3–3	4	3–4	4–4	4	4–4	4–4	**<0.001** ^**a**^	**0.020** ^**a**^	**<0.001** ^**a**^
PEMAT-A	6	2-12	5–9	5	1–12	4.45–7	5	2–8	4–6	**0.008** ^**a**^	**0.040** ^**a**^	**<0.001** ^**a**^
VIQI-sum	13	6–18	12–15	15	10–19	14–17	15	10–20	14–16	**<0.001** ^**a**^	0.588^a^	**<0.001** ^**a**^
VIQI-1	2	1–4	2–3	3	1–5	2–3	4	1–5	3–4	**0.001** ^**a**^	**<0.001** ^**a**^	**<0.001** ^**a**^
VIQI-2	4	1–5	3.25–4	5	3–5	4–5	4	2–5	4–5	**<0.001** ^**a**^	0.062^a^	**<0.001** ^**a**^
VIQI-3	3	1–5	2–4	3	1–5	2–4	2	2–5	2–3	0.352^a^	**<0.001** ^**a**^	**0.003** ^**a**^
VIQI-4	4	2–5	3–4	5	2–5	5–5	5	3–5	5–5	**<0.001** ^**a**^	0.905^a^	**<0.001** ^**a**^
GQS	3	1–5	3–4	5	2–5	4–5	5	3–5	4–5	**<0.001** ^**a**^	0.740^a^	**<0.001** ^**a**^
mDISCERN-sum	3	1–5	3–3	3	3–5	3–3	3	3–4	3–3	0.357^a^	**0.042** ^**a**^	0.647^a^
mDISCERN-1	96 (96.7%)			74 (100%)			99 (100%)			0.507^d^	-	0.246^d^
mDISCERN-2	97 (97.8%)			74 (100%)			99 (100%)			1.000^d^	-	0.497^d^
mDISCERN-3	87 (87.9%)			74 (100%)			97 (98.0%)			**0.008** ^**c**^	0.508^d^	**0.009** ^**b**^
mDISCERN-4	20 (20.2%)			15 (20.3%)			7 (7.1%)			0.982^b^	**0.010** ^**b**^	**0.006** ^**b**^
mDISCERN-5	15 (15.1%)			1 (1.4%)			4 (4.0%)			**0.002** ^**b**^	0.558^c^	**0.007** ^**b**^

P_(Y-B)_: YouTube versus Bilibili; P_(B-T)_: Bilibili versus TikTok; P_(Y-T)_: YouTube versus TikTok. Bold text means the *p*-value < 0.05.

a Mann-Whitney U test.

b Chi-squared test.

c Continuity correction.

d Fisher’s exact test.

**Table 5:** Quality comparison between the videos uploaded by professionals and non-professionals.

Scores	Professionals (N_1_ = 263)			Non-professionals (N_2_ = 8)			*p*-value
	*M*	Min–Max	P25-P75	*M*	Min–Max	P25-P75	
PEMAT-T	9	4–16	8–11	9	5–13	6.5–10	0.317^a^
PEMAT-A	4	2–4	3–4	4	3–4	3.75–4	0.290 ^a^
PEMAT-U	5	1–12	4.5–7	5.5	1–9	2.75–6	0.618^a^
VIQI-1	3	1–5	2–4	3	1–4	1.75–3.25	0.716^a^
VIQI-2	4	1–5	4–5	4	2–5	3.75–5	0.539^a^
VIQI-3	3	1–5	2–4	3.5	1–5	2–4.25	0.453^a^
VIQI-4	5	3–5	4–5	4	2–5	3.5–5	0.155^a^
VIQI-sum	15	8–20	13–16	14	6–19	11.75–17	0.762^a^
GQS	4	1–5	3–5	3.5	2–5	2.75–5	0.310 ^a^
mDISCERN-sum	3	1–5	3–3	3	2–4	2.75–3	0.170^a^
mDISCERN-1	260 (98.9%)			8 (100.0%)			1.000^d^
mDISCERN-2	260 (98.9%)			8 (100.0%)			1.000^d^
mDISCERN-3	248 (94.3%)			6 (75.0%)			0.083^d^
mDISCERN-4	41 (15.6%)			1 (12.5%)			1.000^d^
mDISCERN-5	20 (7.6%)			0			1.000^d^

Bold text means the *p*-value < 0.05.

a Mann-Whitney U test.

d Fisher’s exact test.

### 3.5 Correlation between video quality and audience engagement

**Figure 7:**
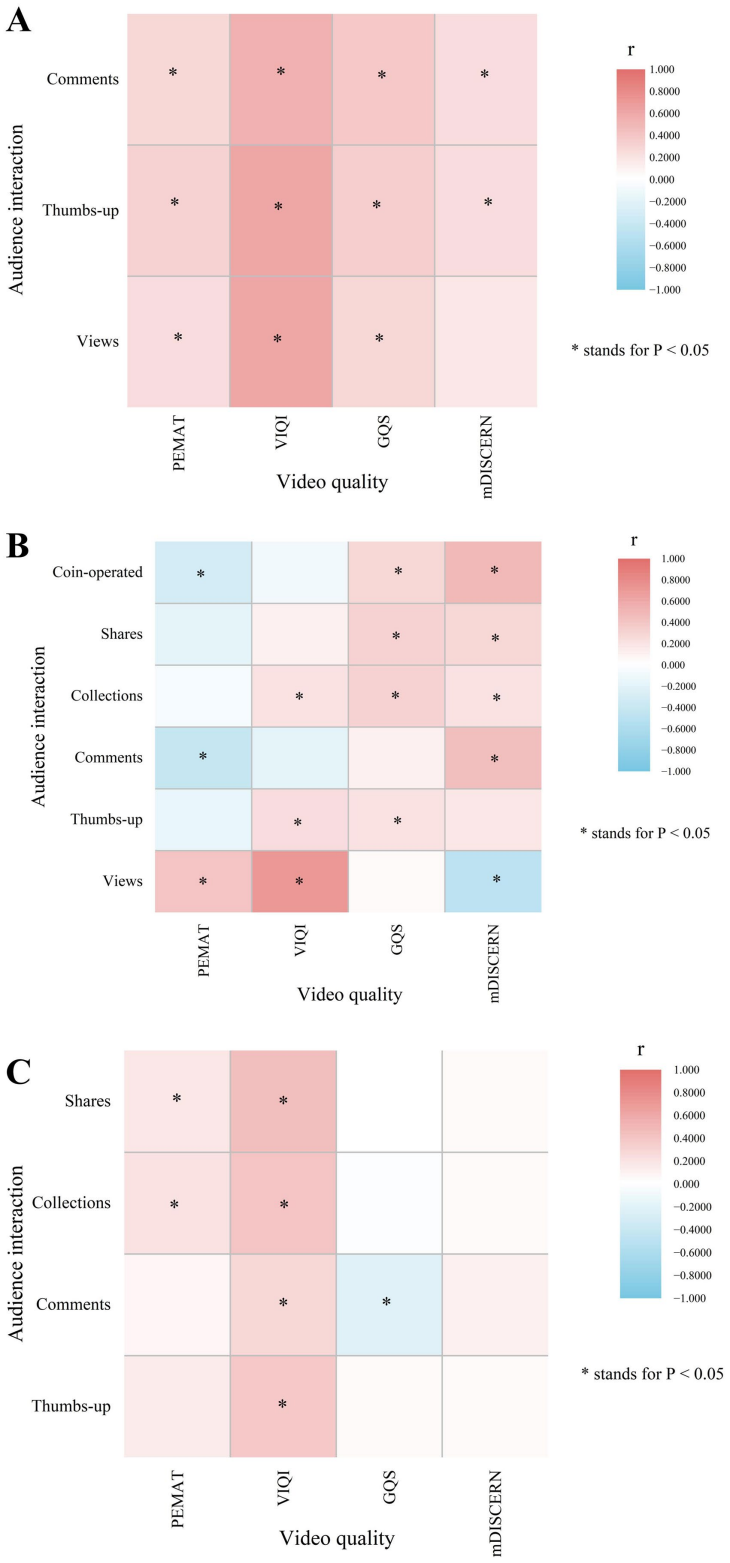
Spearman correlation between video quality and audience interaction on YouTube/ Bilibili/ TikTok. (**A** data source YouTube; **B** data source Bilibili; **C** data source TikTok).

Video quality and audience engagement did not significantly correlate (Table 6; Figure 7). Correlation studies showed that Bilibili’s Views and VIQI had the highest index (0.701, *p* < 0.001), suggesting a strong connection with audience support actions.

**Table 6:** Spearman correlation between video quality and audience interaction on YouTube/ Bilibili/ TikTok.

*r*, *p*-value	YouTube (N_1_ = 98)				Bilibili (N_2_ = 74)				TikTok (N_3_ = 99)			
	PEMAT	VIQI	GQS	mDISCERN	PEMAT	VIQI	GQS	mDISCERN	PEMAT	VIQI	GQS	mDISCERN
Views	0.256, **0.011**	0.605, **<0.001**	0.267, **0.008**	0.191, 0.059	0.421, **<0.001**	0.701, **<0.001**	0.056, 0.579	−0.483, **<0.001**	-	-	-	-
Thumbs-up	0.318, **0.001**	0.607, **<0.001**	0.341, **<0.001**	0.257, **0.011**	−0.144, 0.156	0.238, **0.017**	0.233, **0.020**	0.175, 0.083	0.152, 0.133	0.392, **<0.001**	0.054, 0.596	0.064, 0.532
Comments^a^	0.273, **0.006**	0.560, **<0.001**	0.378, **<0.001**	0.265, **0.016**	−0.424, **<0.001**	−0.172, 0.088	0.108, 0.285	0.435, **<0.001**	0.072, 0.478	0.299, **0.003**	−0.221, **0.028**	0.117, 0.248
Collections	-	-	-	-	−0.065, 0.525	0.201, **0.046**	0.315, **0.002**	0.227, **0.024**	0.205, **0.041**	0.405, **<0.001**	−0.017, 0.868	0.064, 0.530
Shares	-	-	-	-	−0.185, 0.066	0.106, 0.295	0.312, **0.001**	0.284, **0.004**	0.199, **0.049**	0.452, **<0.001**	0.015, 0.884	0.052, 0.610
Coin-operated	-	-	-	-	−0.317, **0.001**	−0.067, 0.508	0.286, **0.004**	0.500, **<0.001**	-	-	-	-

Bold text means the *p*-value < 0.05.

|*r*| ≤ 0.2 no relationship; 0.2 < |*r*| ≤ 0.4 weak relationship; 0.4 < |*r*| ≤ 0.6 moderate relationship; 0.6 < |*r*| ≤ 0.8 strong relationship; |*r*| > 0.8 very strong relationship.

a Excluded: 16 videos on YouTube turned off the function of comments.

Approximately, most of the quality assessment results revealed a mild to moderate positive connection with audience engagement. In contrast to YouTube, Bilibili and TikTok showed varied degrees of negative association between some quality and engagement metrics. PEMAT scores showed a negative link with all audience interaction measures in Bilibili, with the exception of a positive correlation with Views. In Bilibili, on the other hand, VIQI had a negative correlation with Coin-operated and Comments. Surprisingly though, GQS was negatively connected with Comments and Collections on TikTok, while Views and mDISCERN displayed the most negative link in Bilibili (*r* = −0.483, *p* < 0.001).

## 4 Discussion

To identify statistically significant interplatform differences and elucidate variations in audience demographics, we analyzed both long-form (e.g., YouTube, Bilibili) and short-form (e.g., TikTok) video platforms. We employed a comprehensive propensity score matching analysis to delineate the distinctive features of YouTube, Bilibili, and TikTok. Using four distinct scoring methodologies, we derived the following detailed conclusions. This analysis yielded four principal findings: First, significant disparities in content distribution and quality were observed across platforms, with YouTube generally hosting higher-quality productions. Second, uploader identity and platform certification mechanisms significantly influence perceived content reliability. Third, video formats and thematic preferences exhibit substantial regional and cultural variations. Finally, we found no strong correlation between audience interaction metrics and objective video quality measures. Our findings underscore that algorithmic curation, professional certification, cultural context, and user engagement collectively shape video quality and dissemination efficacy. Specifically, platform architecture dictates content format, algorithmic filtering governs content dissemination, professional involvement enhances information quality, while user engagement often diverges from clinical value. A comprehensive summary of these key findings is shown in Table 7.

**Table 7:** Summary of key findings on video quality and engagement across platforms.

Key finding	Data support	Interpretation
No significant difference in video quality between professional and non-professional uploaders	Table 5: All *p* > 0.05 for PEMAT, VIQI, GQS, mDISCERN	Professional credentials do not guarantee higher-quality public health communication.
No major inter-platform differences in information reliability (mDISCERN)	Table 4: mDISCERN-sum scores similar across platforms (*p* > 0.05)	All platforms face similar challenges in conveying reliable medical information.
Weak-to-moderate correlation between engagement and quality metrics	Table 6: Most *r*-values between 0.2–0.4; highest correlation: Bilibili Views vs. VIQI (*r* = 0.701, *p* < 0.001)	Audience engagement is poorly predictive of video quality; high views ≠ high quality.
YouTube leads in comprehensibility and actionability (PEMAT)	Table 4: PEMAT-T and PEMAT-A significantly higher on YouTube (*p* < 0.001)	YouTube videos are more structured and easier to understand and act upon.
Bilibili and TikTok lead in production quality (VIQI, GQS)	Table 4: VIQI and GQS significantly higher on Bilibili and TikTok (*p* < 0.001)	Short-form platforms excel in visual and production quality, but may lack depth.
TikTok has highest engagement despite shortest videos	Table 1: Median length = 114 s; highest likes, comments, shares	Algorithmic promotion and format favor high interaction, not necessarily quality.

Social media, informational videos, and online platforms have become increasingly prominent sources of information. The prevalence of video platforms continues to grow, driven by a public preference for visually presented information over text-based or auditory formats. Notwithstanding the limitations in public health communication, video platforms can augment public health education by assisting medical personnel in improving prescreening efficacy.

Recent studies have investigated video content related to asthma (19), pulmonary rehabilitation (20), inspiratory muscle training (21), COPD (22, 23), allergies (24), and general respiratory health on platforms like YouTube and TikTok. However, these studies have consistently identified significant content quality issues, including inconsistent quality across platforms, the absence of standardized evaluation methodologies, and suboptimal presentation of medical information. Although these video tools show promise for health education initiatives, concerns persist among medical professionals regarding content authenticity and potential misinformation. To ensure reliability and educational value, greater involvement of certified healthcare professionals in the creation and review of medical video content is imperative (25, 26).

Beyond the challenges associated with radiological detection, PNs pose a significant public health threat due to the neglect experienced by a substantial proportion of patients (27). Publications on PN-related video content remain scarce, with the exception of Han et al.’s (28) study. However, that study was limited to an analysis of the top 30 videos on a single Chinese social media platform and did not perform any correlation analysis on video content or quality metrics. Our study is situated within the context of the rapid proliferation of video platforms in contemporary society (29). We implemented a multi-platform (covering domestic and international sites), multi-perspective, and multi-dimensional quality assessment framework. Furthermore, correlation analyses were conducted to examine relationships between key variables.

### 4.1 Significant interplatform disparities in content distribution and quality

YouTube consistently demonstrated superior video quality, particularly in structure and clarity as measured by PEMAT, although its content predominantly focused on examination and diagnosis. In contrast, Bilibili and TikTok offered broader coverage of etiology, prevention, and treatment, but with greater quality variability and occasional inaccuracies. The short-form nature of TikTok content often resulted in superficial coverage of topics, and some videos exaggerated health anxieties to attract views, thereby compromising educational utility.

Video platforms utilize big data analytics to track user behavior patterns, adjusting their algorithms to influence video length and content relevance. Content relevance is fundamentally determined by the specific screening mechanisms employed by each platform. YouTube, which pioneered video sharing in 2005, processes billions of uploads and views daily, hosting diverse content ranging from disease information to disaster reports and personal narratives (9). However, its default “relevance” search function has significant shortcomings: medical content lacks standardized classification and quality assessment systems, search algorithms are suboptimally calibrated, and video review processes lack rigorous oversight (30). TikTok (10) and Bilibili (11) employ complex algorithms that integrate user profiles, search relevance, and interaction patterns to recommend content. However, effectively managing personalized multi-dimensional learning models and trending topics remains challenging. For instance, our analysis revealed that some videos devoid of practical health information gained disproportionate popularity in search results. These videos frequently depict patients experiencing heightened anxiety following chest CT scans that reveal lung nodules, often portraying accompanying emotional distress and depressive symptoms. This suggests that some creators prioritize viewership over all else, disregarding the potential utility of their content for health promotion. Currently popular topics (e.g., celebrities, variety shows, commerce) may be overshadowed by such content. This phenomenon is commonly reported in studies across various disease contexts (10, 31).

From a public health perspective, this phenomenon can influence public perceptions and health behaviors and may exacerbate health misconceptions or anxiety. For instance, although most lung nodules are benign, one-sided or exaggerated content can induce unnecessary panic. Conversely, insufficient dissemination of high-quality information may delay early screening among high-risk groups. According to current clinical guidelines, the probability of malignancy is less than 1% for nodules smaller than 6 mm in diameter, and 1–2% for those measuring 6–8 mm (3). For nodules measuring 6–8 mm, follow-up chest CT scans every 6–12 months are recommended (3). The specific follow-up interval should be individualized based on a comprehensive assessment, including the patient’s risk factors, imaging features suggestive of malignancy, the clinician’s judgment, and patient preferences. For solid nodules larger than 8 mm, management strategies depend on three key factors: (1) the predicted probability of malignancy, (2) the presence of comorbidities (e.g., coronary heart disease or COPD), and (3) patient preferences. Current management options include surgical resection, non-surgical biopsy (e.g., bronchoscopy or transthoracic needle biopsy), positron emission tomography–computed tomography (PET-CT), or active surveillance with serial CT scans to monitor for growth (1, 4). For part-solid nodules, the size of the solid component directly informs management strategy, with larger solid components indicating a higher malignancy risk. Persistent pure ground-glass nodules exceeding 10 mm in diameter have a 10–50% probability of malignant transformation (32). Notably, pure ground-glass nodules typically exhibit slow growth even if malignant. The sensitivity of current diagnostic techniques (e.g., transthoracic biopsy or bronchoscopy) for lung cancer ranges from 70 to 90% (24, 32).

### 4.2 Uploader identity and authentication mechanisms influence content reliability

Although certification methods vary across platforms, independently verifying uploader credentials can help prevent the dissemination of low-quality medical information. Multiple studies indicate that TikTok enforces strict qualification reviews, permitting only chief physicians, associate chief physicians, and registered residents from tertiary Grade A hospitals to use the professional title “doctor” (17, 33). The platform’s algorithm prioritizes videos with high like counts, which can narrow the gap between popularity and perceived quality (34). Consequently, even with stringent certification standards, the correlation between a video’s popularity and its actual quality is not guaranteed. In contrast, Bilibili employs more lenient review standards, allowing grassroots doctors, medical students, and even general users to upload content. This approach diversifies perspectives but also introduces risks to information reliability (25). The public increasingly relies on social media to access health information, encompassing topics from disease surveillance and health education to behavioral change, professional development, and doctor-patient communication (35). Thus, we recommend that all healthcare professionals actively pursue platform certification. Certification can enhance public trust through greater transparency regarding medical expertise. Additionally, certified users often receive greater platform support and visibility. Recent studies on online medical consultation platforms further emphasize the importance of proper incentive structures and team-based collaborations among physicians, which not only improve service quality but also enhance patient satisfaction and trust (36, 37). These findings suggest that platforms could benefit from designing mechanisms that promote collaborative content creation and knowledge sharing among certified professionals. TCM has recently garnered significant attention in disease diagnosis and treatment (38). However, the proportion of TCM practitioners specializing in PNs remains low. Our analysis reveals limited TCM-focused content on PNs within TikTok and Bilibili. Despite this limited presence, some content creators are exploring TCM’s role in managing PNs and innovatively incorporating it, sometimes alongside other diseases, into their diagnostic and therapeutic discussions (10). Therefore, engaging more professional TCM practitioners is crucial to enhancing the quality and representativeness of TCM-related content on these platforms.

Notably, no significant difference was observed in overall video quality between professional and non-professional creators (all *p* > 0.05; see Table 5). This finding challenges the conventional reliance on uploader credentials as a sole proxy for content reliability, demonstrating that professional qualifications do not automatically translate into effective public communication. Similarly, mDISCERN scores showed no significant inter-platform differences, indicating that major platforms share common challenges in conveying medical information reliability. Thus, platforms should implement risk warnings for potentially misleading content (e.g., flagging “controversial viewpoints”) and bolster their accountability. Concurrently, medical schools have incorporated health communication courses to foster interdisciplinary “medicine + communication” skills (39). They also encourage physicians to collaborate with media professionals to translate specialized knowledge into engaging formats, as exemplified by initiatives like the “Medical Science Popularization Alliance” at United Hospital. In conclusion, this study highlights three key recommendations: (1) the public should cultivate critical thinking and diversify information sources; (2) platforms must optimize review mechanisms and balance traffic incentives with social responsibility; and (3) healthcare practitioners should adapt to the digital media landscape and enhance their communication skills.

### 4.3 Regional and cultural variations in video format and thematic preferences

Significant differences exist in both the breadth and focus of topics between YouTube and the Chinese platforms (TikTok, Bilibili). YouTube content focuses predominantly on examination and diagnosis, whereas etiology, prevention, and treatment are the most prevalent topics on TikTok and Bilibili. This preference aligns with a “prevention-first” health philosophy prevalent in Chinese culture (40), where understanding disease origins is valued for preventing progression. Content on Chinese platforms (TikTok, Bilibili) frequently employs monologs and medical scene imagery, emphasizing simplicity and emotional resonance. In contrast, YouTube often utilizes classroom-style or PowerPoint presentations, prioritizing structural clarity and objectivity. Furthermore, medical scene imagery was identified as a particularly effective format for disseminating information on TikTok. Given the diversity of creators and topics, there is a pressing need for platforms and uploaders to integrate multimodal formats to enhance the accessibility and comprehensibility of medical information.

### 4.4 Weak correlation between audience engagement and video quality

With the exception of Bilibili, where view count significantly correlated with VIQI (*r* = 0.701, *p* < 0.001), overall user engagement metrics (e.g., likes, comments, and shares) demonstrated only weak-to-moderate correlations with objective quality measures. This finding is consistent with earlier studies on health science video consumption (41), which suggest that most viewers cannot accurately assess the professional quality of health content, leading to the weak correlations typically observed between engagement and quality (17). This phenomenon can be attributed to several platform-specific factors: (1) cultural and behavioral user group differences, where a preference for entertainment-oriented content creates a “high quality, low interaction” paradox; (2) symbolic interaction behaviors that contrast with the “low quality, high interaction” pattern; and (3) public preference for fragmented viewing, compounded by algorithms that prioritize “high-stimulus” content (e.g., suspenseful thumbnails, rapid editing). The logical rigor inherent to professional medical information is often incompatible with the ultra-short video format. Furthermore, the reflective engagement required for understanding professional medical content fundamentally conflicts with platform algorithms optimized for “community weighting” and “completion rate.” This discrepancy creates a vicious cycle: passive content consumption (rather than active real-time interaction) leads algorithms to classify these users as “low-activity,” consequently reducing the content’s exposure and recommendations. Moreover, content production strategies are heavily influenced by underlying platform business models. YouTube’s ad revenue-sharing model, closely linked to viewing duration and user retention, incentivizes creators to produce higher-quality content to ensure stable income. In contrast, the diversified monetization strategies of TikTok and Bilibili often prioritize traffic-driven content, which can compromise quality. This analysis suggests that the positive correlation between quality and user stickiness on YouTube stems from a synergy of “deep content ecosystem + quality-aligned algorithms + user value recognition.” These findings align with broader research into the acceptance of mHealth services, which highlights the mediating role of perceived usefulness and user attitude in technology adoption (42), as well as the influence of service characteristics on continuous engagement (43). Conversely, the challenges observed on TikTok and Bilibili reflect systemic contradictions between “fragmented consumption + emotion-driven interaction + traffic-optimized algorithms.”

These findings reveal fundamental structural tensions in health communication on commercial video platforms, encompassing conflicts between professional rigor and algorithmic engagement, between cultural preferences and universal standards, and between creator credibility and communicative effectiveness. If unaddressed, these disparities risk exacerbating public misinformation and health anxiety, particularly in clinically nuanced areas like PNs.

Therefore, coordinated multi-stakeholder efforts are required to enhance the quality and accessibility of medical video content. Improving the reliability of online health information is not merely an issue of content moderation; it is a foundational step toward leveraging digital technology to optimize the allocation of medical resources and mitigate regional health disparities (44). Platforms should implement more robust review systems, introduce quality-rating schemes or risk warnings, and adjust algorithms to prioritize scientifically accurate content. Healthcare professionals require training in public communication and should collaborate with media specialists to enhance audience engagement without compromising scientific accuracy. Finally, users should be empowered to develop critical media literacy skills, such as cross-referencing information sources and identifying potential biases. From a policy perspective, promoting a sustainable and reliable mHealth market requires coordinated efforts that address both technological infrastructure and user trust (45). Additionally, the disparate effects of physicians’ knowledge-sharing behaviors—whether driven by satisfaction or gratitude—can significantly influence patients’ evaluation of online medical services (46, 47), underscoring the need for platforms to foster genuine and informative interactions.

Theoretically, this study contributes to health communication scholarship by demonstrating how the interplay of platform algorithms, cultural contexts, and creator credentials shapes medical content quality and dissemination. Methodologically, it provides a validated, multi-dimensional framework for evaluating health-related videos. Practically, it offers actionable insights for platforms, content creators, and educators seeking to improve the effectiveness and reliability of digital health communication.

### 4.5 Limitations

Variations in evaluator expertise and assessment capabilities may introduce subjective bias, despite the use of a respiratory specialist team employing four multi-functional assessment tools. The cross-sectional design captures video attributes related to “Pulmonary Nodule” only at a specific point in time. Consequently, dynamic changes in platform algorithms and content update rates may limit the temporal validity of the findings.

The search strategy relied solely on the single keyword “Pulmonary Nodule,” excluding auxiliary information (e.g., user comments, video descriptions). This approach may have omitted relevant content and reduced search comprehensiveness. Finally, analyzing only the top 100 results per platform may inadequately represent the overall content ecosystem. This approach introduces selection bias, as ranking algorithms prioritize high-engagement (though not necessarily high-quality) content, thereby limiting the generalizability of the conclusions.

These limitations highlight the need for future research to employ multi-platform longitudinal monitoring, natural language processing, and user behavior analytics to develop more comprehensive health information quality assessment models.

## 5 Conclusion

This study offers the first thorough cross-sectional evaluation of lung nodule-related videos across three distinct video platforms. It gives trustworthy information for the general public to comprehend the present situation of online videos connected to PNs on Internet platforms. These insights may help platforms, content creators, and the public at large. While short-form video platforms like TikTok have a high index of audience engagement but poor informativeness, long-form video platforms like YouTube and Bilibili are informative but have low traffic. YouTube videos have the best average quality out of the three platforms, but they can still be made better. This study suggests translating and sharing high-quality information between various platforms for interoperability and learning. A growing number of healthcare professionals are being requested to obtain certification and become proficient in video capturing techniques to enhance the quality of PN-related movies and provide high-quality medical information in an intelligible and clear manner. The platform has developed a complex method for evaluating the content’s quality, improving the recommendation system’s weighting elements, and building a more reliable environment for the spread of health information.

## Data Availability

The original contributions presented in the study are included in the article/Supplementary material, further inquiries can be directed to the corresponding authors. The data analyzed in this study (such as video content, user comments, and public metadata) were sourced from the following publicly available online video platforms: https://www.youtube.com; https://www.bilibili.com; https://www.douyin.com.
