# Supplementary material for: Content accuracy and reliability of pulmonary nodule information on social media platforms: a cross-platform study of YouTube, Bilibili, and TikTok
Source: Front Med (Lausanne). 2025 Sep 22;12:1613526. doi: 10.3389/fmed.2025.1613526 (PMC12499355; doi:10.3389/fmed.2025.1613526)
Supplement: Supplementary file 1 [file Table_1.docx]

**1.mDISCERN** Modified DISCERN

The score of each question ranges from 0 to 1. The total score is 0 to 5.

mDISCERN-1 Are the aims clear and achieved?

0-no 1-yes

mDISCERN-2 Are reliable sources of information used?

0-no 1-yes

mDISCERN-3 Is the information presented balanced and unbiased?

0-no 1-yes

mDISCERN-4 Are additional sources of information listed for patient reference?

0-no 1-yes

mDISCERN-5 Are areas of uncertainty mentioned?

1. no 1-yes

A verified rating scale and scoring criteria, the mDISCERN tool is intended to evaluate the quality of medical textual materials and associated videos[1][2]. The mDISCERN tool user's guide states that movies with a total score of 3 are of intermediate quality, those with a score higher than 3 are of good quality, and those with a score lower than 3 are of bad quality. The benefits of mDISCERN include its widespread use, ease of comprehension, speed, and ease of use; but, its limited assessment dimensions and alternatives (yes or no) do not fully meet our needs for video content evaluation. For instance, not all of the questions in the mDISCERN tool ask about video traffic, which is a crucial component in determining the quality of videos.

**2. GQS** Global Quality Score

The total score is 1 to 5.

【Score 1】Poor quality, poor flow of the site, most information missing, not at all useful for patients.

【Score 2】Generally poor quality and poor flow, some information listed but many important topics missing, of very limited use to patients

【Score 3】Moderate quality, suboptimal flow, some important information is adequately discussed but others poorly discussed, somewhat useful for patients

【Score 4】Good quality and generally good flow, most of the relevant information is listed, but some topics not covered, useful for patients

【Score 5】Excellent quality and excellent flow, very useful for patients

Compared to mDISCERN, which considers video traffic, website flow, and user-friendliness, the Global Quality Score (GQS), developed by Singh et al. in 2012, is the most comprehensive, straightforward, and widely applicable assessment method for evaluating the overall quality of videos[3]. We included two more scoring systems as a complement since GQS lacks detailed assessment and the evaluation criteria are quite arbitrary and impossible to measure.

**3. VIQI** Video Information and Quality Index.

The score of each question ranges from 1 to 5. The total score is 5 to 20.

【VIQI 1】 information flow

*The VIQI 1 in our study is according to thumbs-up because the data of thumbs-up are available from all three platforms.

1 ＜10 thumbs-up

2 ＜100 thumbs-up

3 ＜1000 thumbs-up

4 ＜10000 thumbs-up

5 ≥10000 thumbs-up

* If a video turns off the bottom of thumbs-up (very few would do this), we rate its flow by views.

1 ＜100 views

2 ＜1000 views

3 ＜10000 views

4 ＜100000 views

5 ≥100000 views

【VIQI 2】 information accuracy

【VIQI 3】quality (videos including one point for each image, animation, interview, video captions, and summary)

【VIQI 4】 precision (level of coherence between video title and content)

Since the first item on the original scale, "information flow," is not clearly defined, we define it as the quantity of likes or plays and quantify it. The Video Information and Quality Index (VIQI) is a five-point Likert scale used to evaluate video quality[4]. The assessment criteria of "information flow" are quantified by VIQI as an addition to GQS, which expands the scope of evaluation but still has issues with bias and lack of consistency.

**4. PEMAT** Patient education materials assessment tool

TOPIC: CONTENT

1.The material makes its purpose completely clear. (Disagree = 0, Agree = 1)**

TOPIC: WORD CHOICE & STYLE
3. The material uses common, everyday language. (Disagree = 0, Agree = 1)
4. Medical terms are used only to familiarize the audience with terminology, and are defined when used. (Disagree = 0, Agree = 1)
5. The material uses the active voice. (Disagree = 0, Agree = 1)

TOPIC: ORGANIZATION
8. The material divides information into short sections. (Disagree = 0, Agree = 1; Not applicable if the material is very short)
9. The sections have informative titles. (Disagree = 0, Agree = 1; Not applicable if the material is very short)
10. The material presents information in a logical order. (Disagree = 0, Agree = 1)
11. The material provides a summary. (Disagree = 0, Agree = 1; Not applicable if the video is very short)

TOPIC: LAYOUT & DESIGN
12. The material uses visual cues (e.g., arrows, boxes, bullet points, bold text, larger fonts, highlighting) to draw attention. (Disagree = 0, Agree = 1; Not applicable for video)
13. The text on the screen is easy to read. (Disagree = 0, Agree = 1; Not applicable if there is no text or all text is narrated)
14. The material allows users to clearly hear the words (e.g., the speaking rate is not too fast, and the speech is not mumbled). (Disagree = 0, Agree = 1; Not applicable if there is no narration)

TOPIC: USE OF VISUAL AIDS
18. The illustrations and photos used in the material are clear and not cluttered. (Disagree = 0, Agree = 1; Not applicable if there are no visual aids)
19. The material uses simple tables with brief and clear row and column headings. (Disagree = 0, Agree = 1; Not applicable if there are no tables)

TOPIC: ACTIONABILITY
20. The material clearly indicates at least one action that the user can take. (Disagree = 0, Agree = 1)
21. The material addresses the user directly when describing actions. (Disagree = 0, Agree = 1)
22. The material breaks down any actions into manageable and clear steps. (Disagree = 0, Agree = 1)
25. The material explains how to use charts, graphs, tables, or diagrams to take action. (Disagree = 0, Agree = 1; Not applicable if there are no charts, graphs, tables, or diagrams)

CONTENT, ORGANIZATION, LAYOUT & DESIGN, USE OF VISUAL AIDS, and ACTIONABILITY are the main qualities evaluated by the PEMAT scale, a method designed to evaluate the comprehensibility and actionability of patient education materials[5]. To meet the evaluation of this movie, we simplified and streamlined a few of the tests.The Agency for Healthcare Research and Quality created and released PEMAT in 2014, and it has now been shown to be a very accurate, thorough, standardized, and objective evaluation tool that is used extensively worldwide[6]. PEMAT-T is the assessment tool's general framework; PEMAT-U is the assessment material's comprehensibility; and PEMAT-A is the assessment material's operability. The one-sidedness, standardization, and lack of objectivity of the other three approaches are somewhat addressed by PEMAT; however, because of its unique features, it also has issues with scoring process complexity and universality (the requirement to modify the assessment criteria based on the content to be evaluated). Owing to its unique features, it also has a difficult scoring procedure and is not universal (the evaluation criteria must be modified based on the content being evaluated).

**References:**

1. ReFaey K, Tripathi S, Yoon JW, Justice J, Kerezoudis P, Parney IF, Bendok BR, Chaichana KL, Quiñones-Hinojosa A. The reliability of YouTube videos in patients education for Glioblastoma Treatment. J Clin Neurosci. 2018 Sep;55:1-4. doi: 10.1016/j.jocn.2018.07.001. Epub 2018 Jul 31. PMID: 30075980.
2. Charnock D, Shepperd S, Needham G, Gann R. DISCERN: an instrument for judging the quality of written consumer health information on treatment choices. J Epidemiol Community Health. 1999 Feb;53(2):105-11. doi: 10.1136/jech.53.2.105. PMID: 10396471; PMCID: PMC1756830.
3. Bernard A, Langille M, Hughes S, Rose C, Leddin D, Veldhuyzen van Zanten S. A systematic review of patient inflammatory bowel disease information resources on the World Wide Web. Am J Gastroenterol. 2007 Sep;102(9):2070-7. doi: 10.1111/j.1572-0241.2007.01325.x
4. Tamošiūnaitė I, Vasiliauskas A, Dindaroğlu F. Does YouTube provide adequate information about orthodontic pain? Angle Orthod. 2023 Jul 1;93(4):403-408. doi: 10.2319/072822-527.1. PMID: 36820821; PMCID: PMC10294584.
5. Yeung A, Ng E, Abi-Jaoude E. TikTok and Attention-Deficit/Hyperactivity Disorder: A Cross-Sectional Study of Social Media Content Quality. Can J Psychiatry. 2022 Dec;67(12):899-906. doi: 10.1177/07067437221082854. Epub 2022 Feb 23. PMID: 35196157; PMCID: PMC9659797.
6. Shoemaker SJ, Wolf MS, Brach C. Development of the Patient Education Materials Assessment Tool (PEMAT): a new measure of understandability and actionability for print and audiovisual patient information. Patient Educ Couns. 2014 Sep;96(3):395-403. doi: 10.1016/j.pec.2014.05.027. Epub 2014 Jun 12. PMID: 24973195; PMCID: PMC5085258.
