# Supplementary material for: Content accuracy and reliability of pulmonary nodule information on social media platforms: a cross-platform study of YouTube, Bilibili, and TikTok
Source: Front Med (Lausanne). 2025 Sep 22;12:1613526. doi: 10.3389/fmed.2025.1613526 (PMC12499355; doi:10.3389/fmed.2025.1613526)
Supplement: Supplementary file 2 [file Table_2.docx]

**Details in Assessment Tools**

1. **Certification**

Fulfillment of any of the following conditions is considered certification.

**1.1 YouTube**

There's a gray tick to the right of his ID

For example:


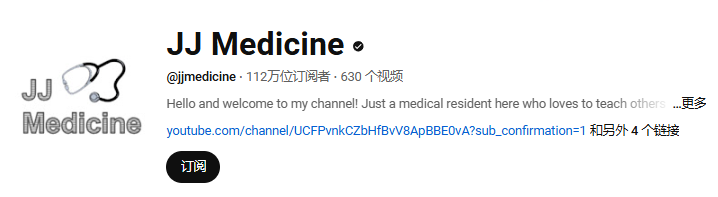


**1.2 Bilibili**

①Yellow Flash:Personal authentication for celebrity/popular accounts.

For example:


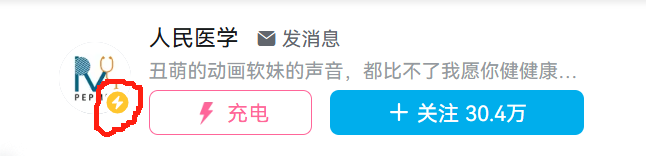


②Blue Flash:Group Certification.

For example:


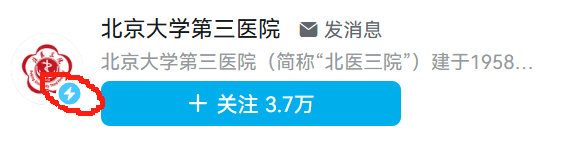


③On Bilibili, anyone with a license to practice medicine and a certificate of employment from any hospital can apply for a “Gray V”.

For example:


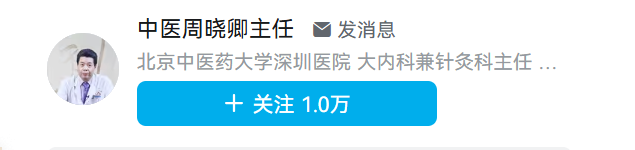


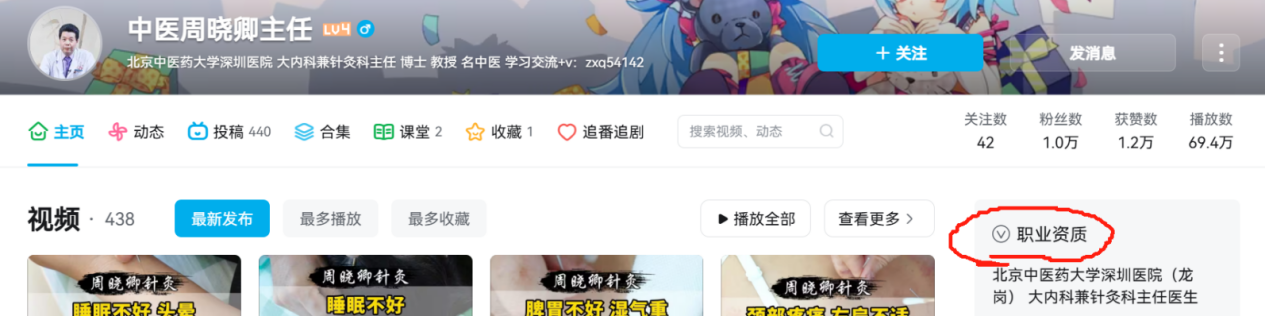


④Theoretically, an account might get both Yellow Flash and Gray V.

For example:


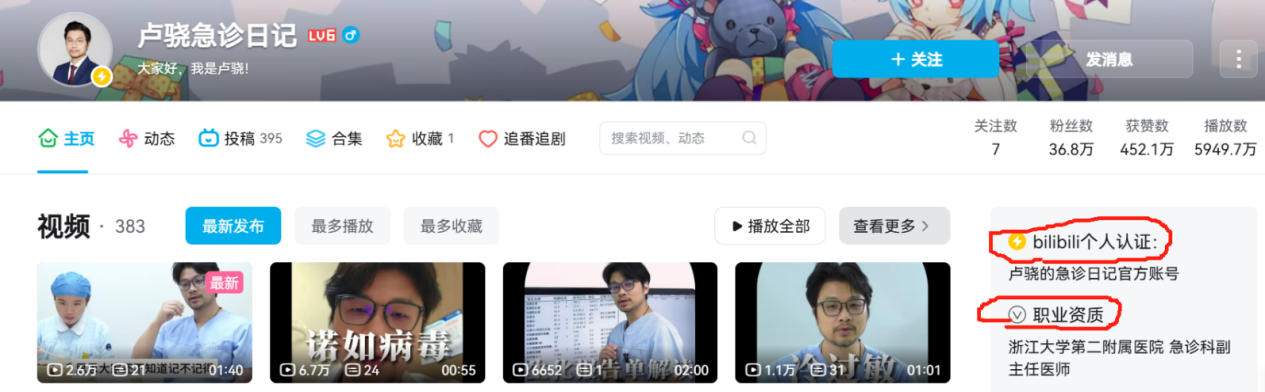


**1.3 TikTok**

①　Yellow V:Personal certification for celebrities/popular accounts/professions.

On TikTok, only attending doctors/associate doctors/chief doctors who work in tertiary and primary hospitals (in China's hospital ranking system, tertiary and primary means the highest level) can apply for grey v. Residents or any doctors who do not work in tertiary and primary hospitals can apply for NOT. NOT uploads health-related videos on TikTok.This stringent certification requirement for doctors comes into effect in June 2023.

For example:


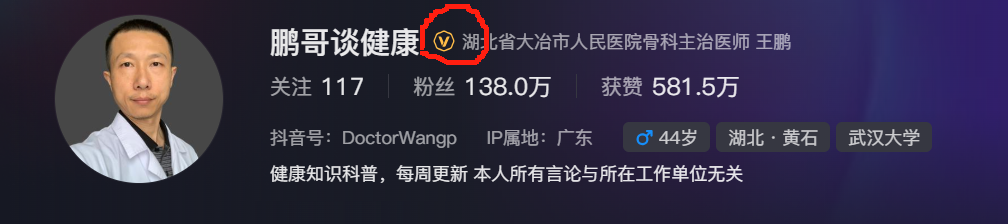


②　Blue V:Group Certification

For example:


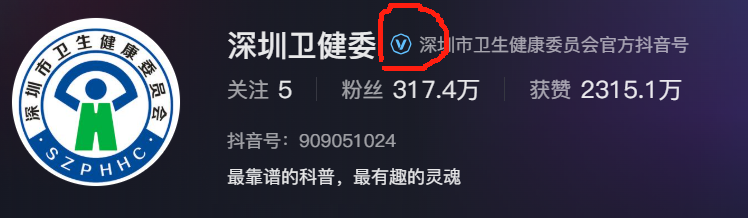


③　Red V:Certification of public accounts licensed to provide Internet news and information services.

For example:


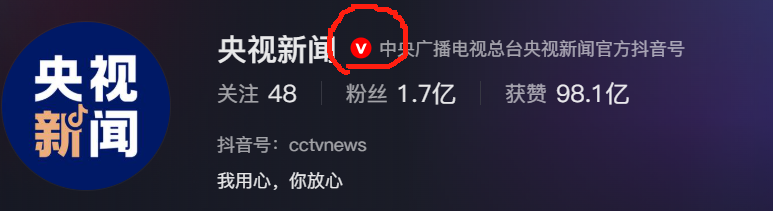


**2. Advertising**

**2.1 YouTube**

On the YouTube browsing page, the term “sponsored” shows that it is an advertising. Since these commercial films are only advertisements, which is how YouTube generates revenue, they had nothing to do with the user's search parameters and were thus excluded from our analysis. Users are aware that they are advertisements without having to click to view them. We "skip" it because of this.

**2.2 Bilibili and TikTok**

Neither TikTok nor Bilibili have ads tagged as "sponsored" videos, in contrast to YouTube.

**2.3 Additional findings**

Our study did not classify a video as an advertising if its main goal is to spread knowledge rather than make money (for example, by demonstrating a product). We can sell electronic blood pressure monitors or portable home blood glucose meters and make a little more money than we can with diabetes or hypertension. However, people with lung nodules have fewer of these diseases compared to those with high blood pressure, diabetes and other prevalent diseases. Most of the videos share diets, teas, and other products. Additionally, the comments area of TikTok and Bilibili videos sometimes include links to items.

**3. Originality**

We'll classify the following as non-original:

①　Other people's watermarks.

②　Absence of tags for uniqueness.

What does "original tag" mean? YouTube and TikTok do not provide this option on Bilibili. The originality tag, for instance, is shown by the red arrow.

For example:


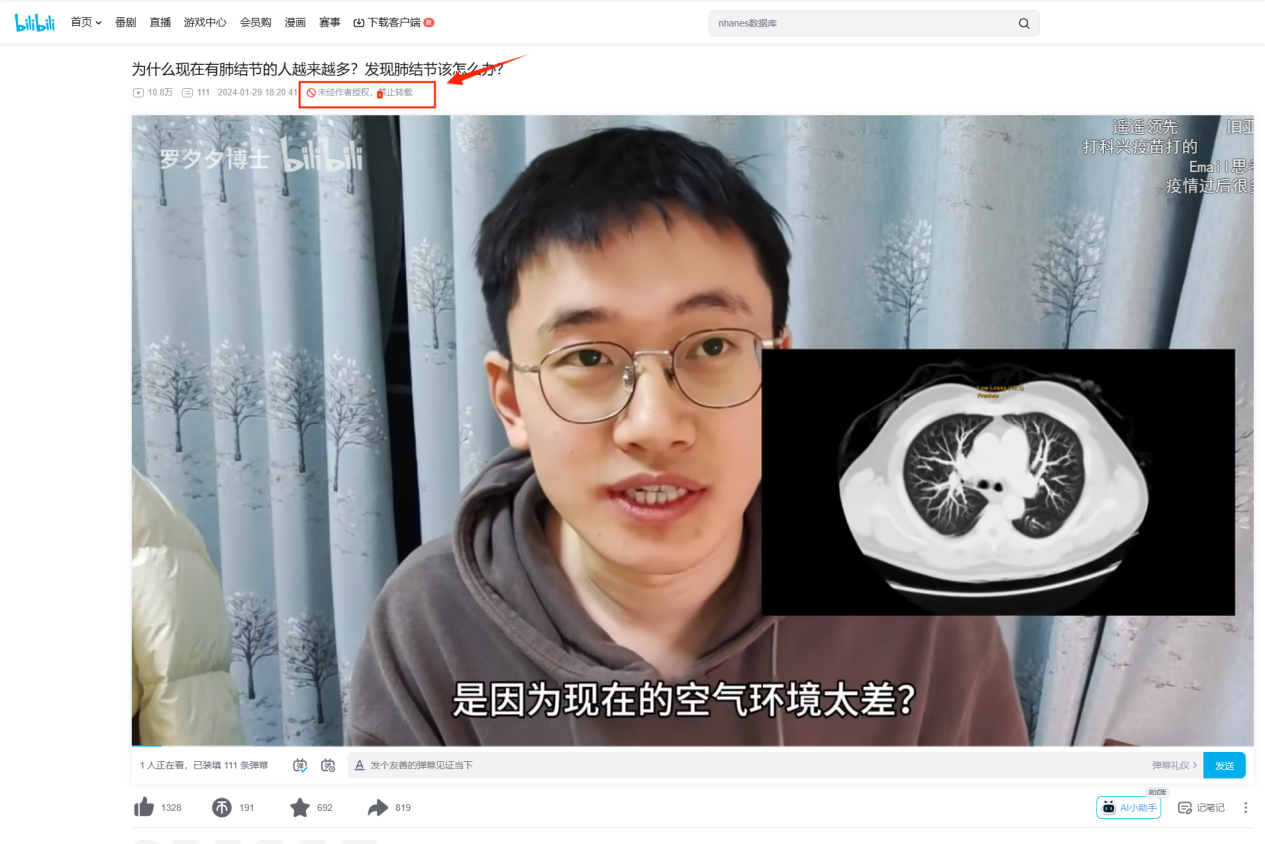


The absence of the original tag is indicated by a red arrow. A yellow arrow indicates that the uploader's ID does not correspond to the name in the video. This indicates that the video is a repost rather than the original.

For example:


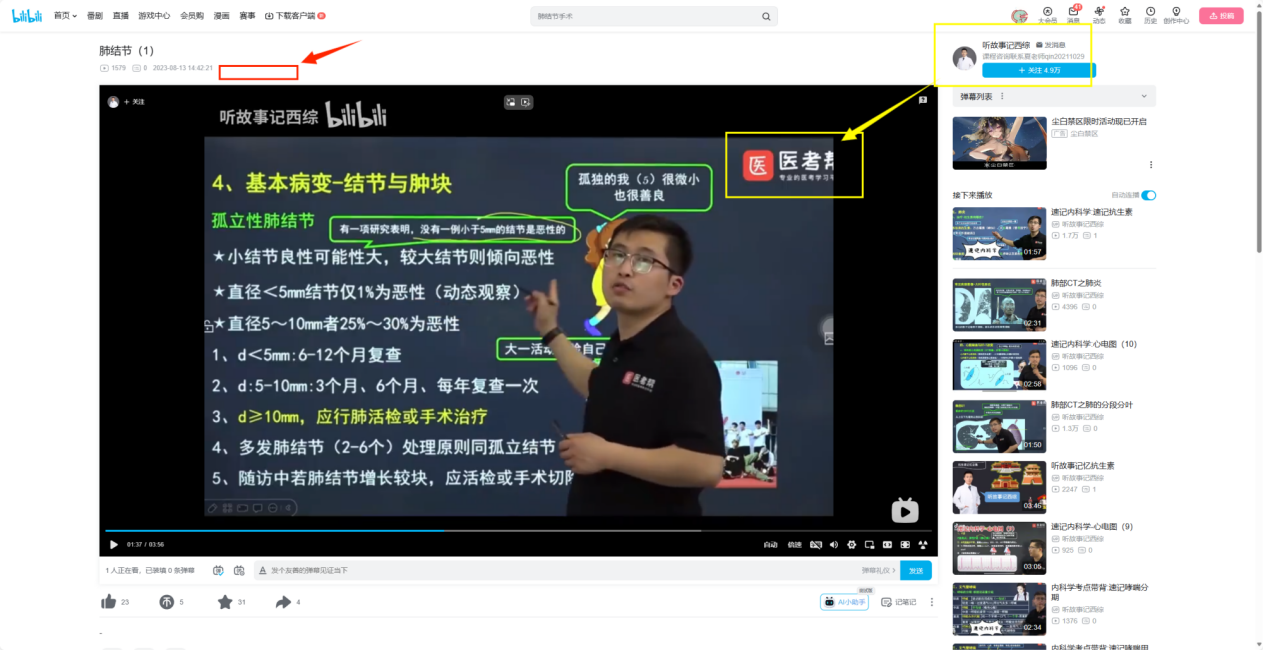


③　Reprints, translations, and crude reprints are not considered original.

Although determining originality is subjective, we have made every effort to make sure that every assessment is fair.

Bilibili has the benefit of being able to play high-quality videos from other platforms, which promotes the spread of information. The user experience may be impacted by the presence of similar films, such as documentaries that have been cut into similar movies of varying durations or English videos on YouTube that have been translated into Chinese and posted by many users.It is necessary to enhance Bilibili's copyright policy in order to balance these benefits and drawbacks.

**4. Inclusion and exclusion criteria**

**4.1 Inclusion criteria**

① Search date: March 1, 2025

② Search Keywords:YouTube “Pulmonary Nodule”, Bilibili and TikTok “肺结节”(Chinese version of “Pulmonary Nodule” in Chinese).

③ Order:Default order, without any filters

④ Published more than 2 weeks ago. (Depending on the services provided by each platform, the data on the number of views and likes in the first week are unstable and do not accurately reflect audience engagement.) After the inclusion criteria, we got all the videos from each platform. However, we had not yet watched the videos, and we did not know whether a particular video was authenticated or not, and whether the content was duplicated or irrelevant. We then moved to the exclusion criteria section.

**4.2 Exclusion Criteria**

① No authentication video. See 1

② Video with commercial advertisement. See 2

③ Non-original video. See 3

④ Similar videos. If two videos are similar (identical or edited from the same source), the video uploaded by the certified account is retained. If neither video is authenticated, the video uploaded first is retained.

⑤ Irrelevant videos. The topics of the videos were categorized as Anatomy, Etiology/Prevention, Pathology, Epidemiology, Symptoms, Screening/Diagnosis, Treatment, and Prognosis. Videos that did not address these topics were considered irrelevant. Or the content of the video was not related to sleep apnea syndrome

**5. Video Shooting Style**

①　Solo narration: Only one person talking in the video, no other scenes.

Example: https://www.bilibili.com/video/BV1Aw4m1f7La/?share_source=copy_web&vd_source=ce72ecae3996e1997a88a0ec8e202dfd

②　Q&A: One person asks a question (or a question written in subtitles) and the other person answers.

Example: https://www.bilibili.com/video/BV1gDHSeBEwA/?share_source=copy_web&vd_source=ce72ecae3996e1997a88a0ec8e202dfd

③　PPT/Class: The video producer uses PowerPoint to present the video, such as in some online medical courses for medical students.

Example: https://www.bilibili.com/video/BV1Kg411b7YV/?share_source=copy_web&vd_source=ce72ecae3996e1997a88a0ec8e202dfd

④　Animation/Motion: The content of the video screen is basically all animation

Example: https://www.bilibili.com/video/BV1ze411q7Sc/?share_source=copy_web&vd_source=ce72ecae3996e1997a88a0ec8e202dfd

⑤　Medical scenarios: Video screen content for real medical scenarios

Example:

https://www.bilibili.com/video/BV1wu4m1A7kS/?share_source=copy_web&vd_source=ce72ecae3996e1997a88a0ec8e202dfd

⑥　TV programs/documentaries: The video is in the form of a documentary

Example: https://www.bilibili.com/video/BV1HH4y127eH/?share_source=copy_web&vd_source=ce72ecae3996e1997a88a0ec8e202dfd

⑦　Other

Example: https://www.bilibili.com/video/BV1mL411R7u3/?share_source=copy_web&vd_source=ce72ecae3996e1997a88a0ec8e202dfd
